# The usefulness of immunohistochemistry for phosphohistone H3 as a prognostic factor in myxoid liposarcoma

**DOI:** 10.1038/s41598-023-31896-y

**Published:** 2023-03-23

**Authors:** Akira Takazawa, Yasuo Yoshimura, Masanori Okamoto, Atsushi Tanaka, Munehisa Kito, Kaoru Aoki, Takeshi Uehara, Jun Takahashi, Hiroyuki Kato, Jun Nakayama

**Affiliations:** 1 Department of Orthopaedic Surgery, Shinshu University School of Medicine, 3-1-1 Asahi, Matsumoto, Nagano 390-8621 Japan; 2 Department of Orthopaedic Surgery, Shinshu Ueda Medical Center, 1-27-21 Midorigaoka, Ueda, Nagano 386-8610 Japan; 3 Rehabilitation Center, Shinshu University Hospital, 3-1-1 Asahi, Matsumoto, Nagano 390-8621 Japan; 4 Department of Applied Physical Therapy, Shinshu University School of Health Sciences, 3-1-1 Asahi, Matsumoto, Nagano 390-8621 Japan; 5 Department of Laboratory Medicine, Shinshu University School of Medicine, 3-1-1 Asahi, Matsumoto, Nagano 390-8621 Japan; 6 Department of Molecular Pathology, Shinshu University School of Medicine, 3-1-1 Asahi, Matsumoto, Nagano 390-8621 Japan

**Keywords:** Cancer, Oncology

## Abstract

Myxoid liposarcoma (MLS) is a common subtype of liposarcoma. Although the prognosis is generally good, there are factors known to be associated with poor prognosis. Accurate indices are important to predict prognosis. We aimed to assess the usefulness of immunohistochemistry for phosphohistone H3 (PHH3) as a potential biomarker in comparison with Ki-67 antigen and other prognostic factors. Twenty-five patients with MLS were evaluated. Age, sex, depth of tumor, tumor size, surgical margin, oncological outcome, histological grade, presence of necrosis, proportion of round cell component (RC%), PHH3 index, and Ki-67 index were examined. Prognostic factors of the examination criteria were statistically analyzed, survival rate analyses were performed using the Kaplan–Meier method, and multivariate analysis was performed using Cox proportional-hazard regression analysis. The number of PHH3-positive tumor cells and the PHH3 and Ki-67 indices demonstrated a statistical correlation with the prognosis of MLS in univariate analysis (*P* < 0.001, *P* < 0.001, *P* = 0.01, respectively). PHH3 index and RC% were significant factors in multivariate analysis (*P* = 0.03, *P* = 0.02). The immunohistochemistry of PHH3 may be associated with prognosis and could serve as a valid criterion of histological grade in MLS.

## Introduction

Soft tissue sarcoma (STS) is a rare malignant neoplasm that accounts for approximately 1% of all malignancies. Larger, deep-seated, and histological high-grade tumors are associated with poor survival. In addition, specific prognostic factors for some subtypes of STS are documented in the literature^1,2^. For instance, our study described the role of several glycans in STS with myxoid substance and reported that chondroitin sulfate synthase 1 (CHSY1) expression was closely associated with their malignant potential^3^. Since myxoid liposarcoma (MLS) is one of the most common subtypes of STS^1,4–6^, the same study examined CHSY1 expression in MLS^3^. The results showed that the frequency of CHSY1 in MLS was 25%, which was lower than that of other histologic types including myxofibrosarcoma, malignant peripheral nerve sheath tumor, and low-grade fibromyxoid sarcoma; however, the expression was limited to those showing round cell morphology indicative of poor patient prognosis. Although previous reports have shown prognostic factors for MLS including tumor size, surgical margin, histological grade, distant metastasis, and proportion of round cell component that has been described as a hypercellularity in the 2020 WHO classification, its pathological prognostic factors remain to be fully defined^7–15^. Therefore, we sought other biomarkers that predict the prognosis of these patients more accurately.

Epigenetic changes in DNA or histone modification have recently been indicated to be related to the malignant transformation of cells. Histone is the core protein of nucleosome that is a fundamental structure of chromatin. The modification of histone tails can diversify the chromatin structure and promote or suppress the proliferation of cells. In particular, the phosphorylation of histone H3 is known to be related to mitosis^16,17^. Phosphohistone H3 (PHH3) is specifically expressed in the G2-M period of the cell cycle, in contrast to Ki-67 antigen which is expressed in every period except G0^18,19^. Accordingly, there are several reports that argue the usefulness of PHH3 as a marker of mitotic count in brain tumor, melanoma, gastrointestinal stromal tumors (GIST), and others^19–25^. Contrariwise, there are very few reports for sarcoma patients.

We focused on the assessment of PHH3 as a new index for the histological grading of MLS. Since the tendency of PHH3 among various subtypes of sarcoma is unknown, we focused our study on a single tumor type. In this study, we investigated the relationship between the number or proportion of PHH3-positive tumor cells and the prognosis in MLS, and we assessed the usefulness of immunohistochemistry for PHH3 as a potential biomarker in comparison with Ki-67 antigen and other prognostic factors.

## Results

### Clinical characteristics of patients

The median age of patients at operation was 53.0 years (range 31–85), of which 15 were males and ten were females. The median follow-up period was 8.2 years (range 1.5–22.3). The median diameter of tumors was 9.0 cm (range 4.4–23.0). The tumors eventually metastasized in five patients, including three patients who were already detected at first visit. The oncological outcomes were continuous disease free (CDF) in 17 patients, alive with disease (AWD) in one, dead of disease (DOD) in five, and dead of other disease (DOOD) in two (Table 1). The outcomes of all patients with metastasis were DOD.

**Table 1:** Clinical characteristics of 25 patients with myxoid liposarcoma.

Characteristic	Number of Pt. or median	Range
Age (years)	53.0	31–85
*Sex*		
Male	15	
Female	10	
Flow-up (years)	8.2	1.5–22.3
Tumor size (cm)	9.0	4.4–23.0
*Outcome*		
CDF	17	
AWD	1	
DOD	5	
DOOD	2	

*CDF* Continuous disease free, *AWD* Alive with disease, *DOD* Dead of disease, *DOOD* Dead of other disease.

Clinicopathological and immunohistochemical data for each individual patient are shown in Supplemental Table 1.

### Statistical analysis of PHH3-positive tumor cells in MLS

**Figure 1:**
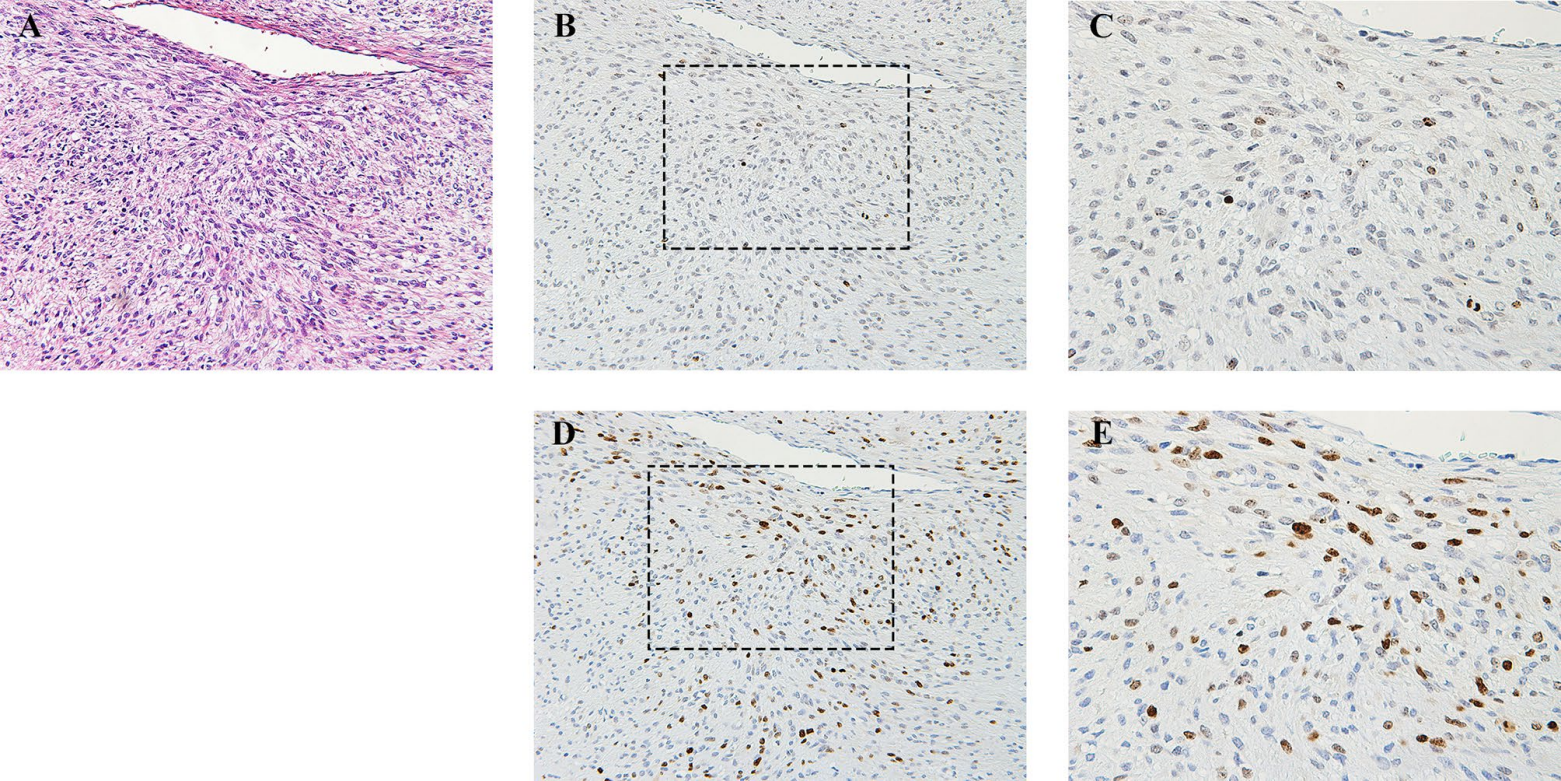
Immunohistochemical expression of PHH3 and Ki-67 in an MLS patient showing the PHH3 index of 2.1% and Ki-67 index of 31.3%. (**A**) H & E (X100), (**B**) PHH3 (X100), (**C**) PHH3 (X200), (**D**) Ki-67 (X100), (**E**) Ki-67 (X200).

**Figure 2:**
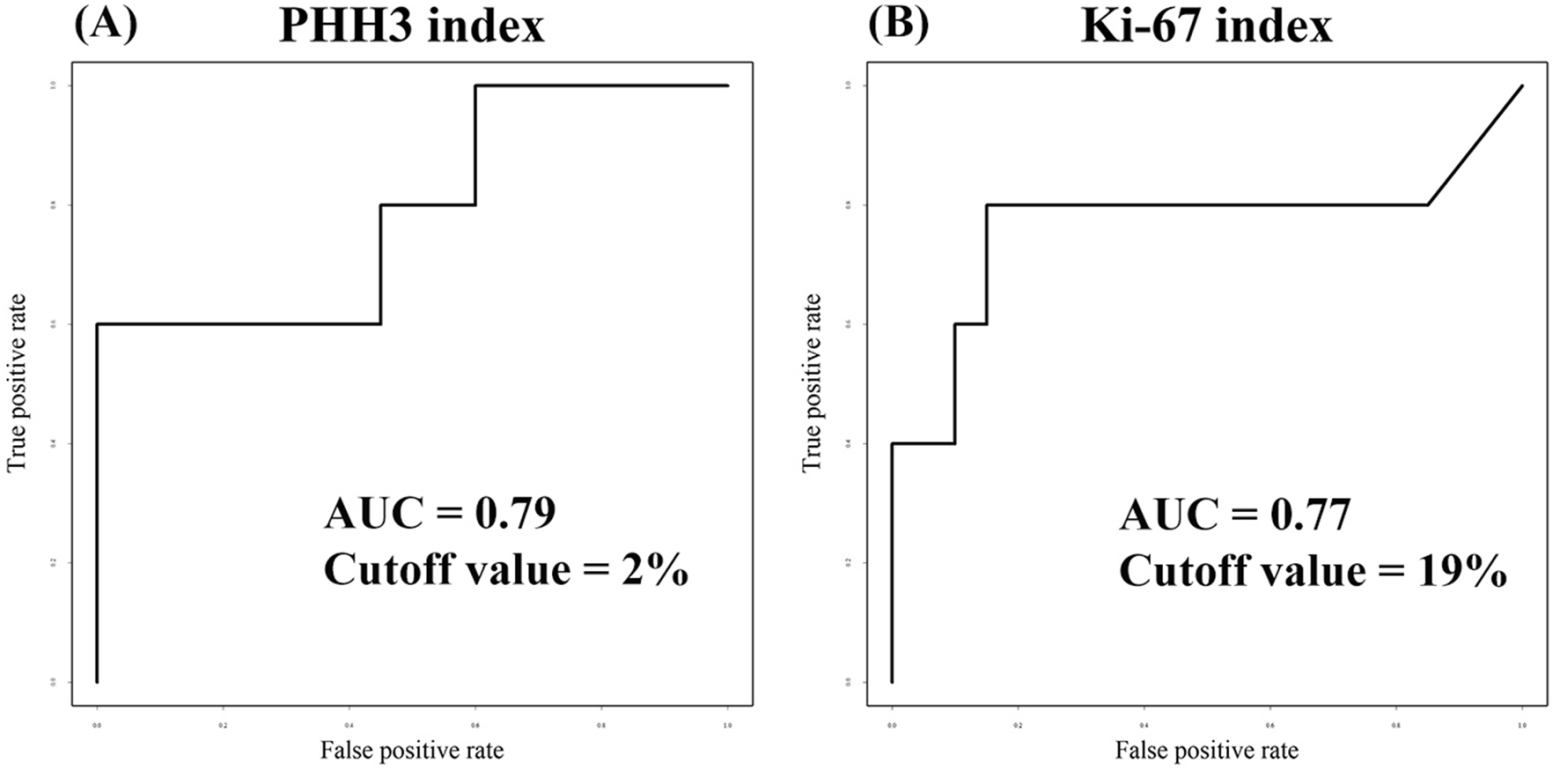
ROC curves of (**A**) PHH3 index and (**B**) Ki-67 index. Each cut-off value of the highest sensitivity and specificity is determined.

**Figure 3:**
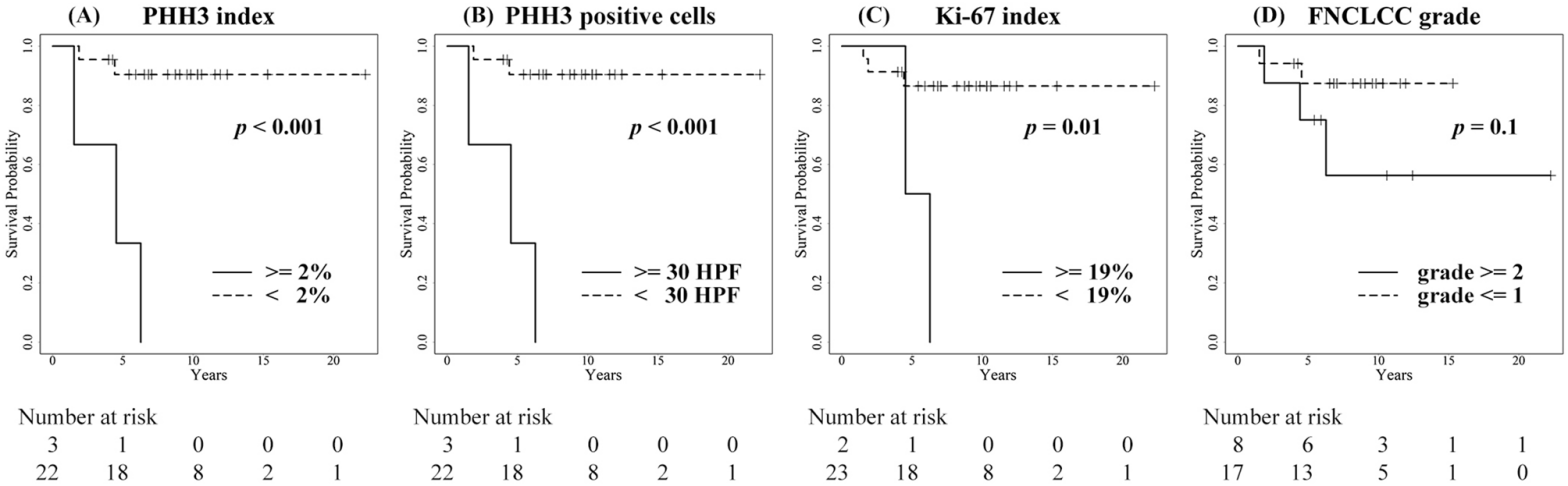
Kaplan–Meier curves of DSS in (**A**) PHH3 index, (**B**) number of PHH3 positive tumor cells, (**C**) Ki-67 index, and (**D**) FNCLCC grade. *P*-values are calculated by Log-rank test.

In PHH3 immunohistochemistry, the number of total tumor cells and positive tumor cells on the hot spots were measured in 10 high power fields (HPF) (Fig. 1). The positive rate of PHH3 was subsequently calculated for consideration as PHH3 index. The median number of PHH3-positive tumor cells was 6 (range 0–45) cells per 10 HPF, and the PHH3 index was 0.3 (range 0.0–10.7) % on immunostained specimens (Table 2). The cut-off values of the highest sensitivity and specificity were determined by the receiver operating characteristic (ROC) curves. For example, the cut-off values of PHH3-positive tumor cells and the PHH3 index were 30 cells per 10 HPF and 2%, respectively (Fig. 2A, Table 2). The differences in disease-specific survival rate (DSS) at cut-off values were calculated by the Kaplan–Meier method (Fig. 3A, B). In the univariate analysis using the Log-rank test, the number of PHH3-positive tumor cells and the PHH3 index demonstrated a statistically significant difference (*P* < 0.001). In both the number of PHH3-positive tumor cells and the PHH3 index, the five-year DSS below and above the cut-off value was 90.4% and 33.3%, respectively (Table 3). In the multivariate analysis using Cox proportional-hazard regression analyses, the PHH3 index was a significant factor (*P* = 0.03) (Table 4).

**Table 2:** Pathological characteristics of surgical specimens and cutoff values from ROC curves.

Characteristic	Median	Range	AUC	Cutoff value
All cells (/10HPF)	2463	283–5522	0.48	
PHH3 positive tumor cells (/10HPF)	6	0–45	0.89	30
Ki-67 positive tumor cells (/10HPF)	54	0–984	0.59	
PHH3 index (%)	0.3	0.0–10.7	0.79	2
Ki-67 index (%)	2.2	0.0–31.3	0.77	19

*PHH3* Phosphohistone H3, *HPF* High power fields, *AUC* Area under the curve, *ROC* Receiver operating characteristic.

**Table 3:** Disease-specific survival rate using the Kaplan–Meier method and univariate analysis using the log-rank test.

Variable	Number of Pt	5-year DSS (%)	*P* value
PHH3 positive tumor cells (/10HPF)			< 0.001
< 30	22	90.4	
≥ 30	3	33.3	
PHH3 index (%)			< 0.001
< 2	22	90.4	
≥ 2	3	33.3	
Ki-67 index (%)			0.01
< 19	23	86.5	
≥ 19	2	50.0	

*PHH3* Phosphohistone H3, *HPF* High power fields.

**Table 4:** Multivariate analysis using the Cox proportional-hazard regression analyses.

Variable	HR	95% CI	*P* value
PHH3 index (%)	1.35	1.03–1.76	0.03
Ki-67 index (%)	1.10	0.98–1.24	0.10
Round cell component (≥ 5%)	42.7	1.93–943	0.02

*PHH3* Phosphohistone H3, *HR* Hazard ratio, *CI* Confidence interval.

### Statistical analysis of Ki-67 in MLS

Ki-67 was measured by the same method with PHH3 for comparison (Fig. 1). The median number of Ki-67-positive tumor cells and the Ki-67 index were 54 (range 0–984) cells per 10 HPF and 2.2 (range 0.0–31.3) %, respectively (Table 2). The cut-off value of the Ki-67 index was determined to be 19% based on the ROC curve (Fig. 2B). The value of Ki-67-positive tumor cells was difficult to determine due to its low area under the curve (AUC) value on the ROC curve. The differences in DSS at the cut-off value were calculated using the Kaplan–Meier method (Fig. 3C), and univariate analysis was performed using the Log-rank test. The Ki-67 index presented a statistically significant difference (*P* = 0.01). The five-year DSS below and above the cut-off value was 86.5% and 50.0% in the Ki-67 index (Table 3). In the multivariate analysis, the Ki-67 index was not a significant factor (*P* = 0.10) (Table 4).

### Correlation between the PHH3 and Ki-67 indices

**Figure 4:**
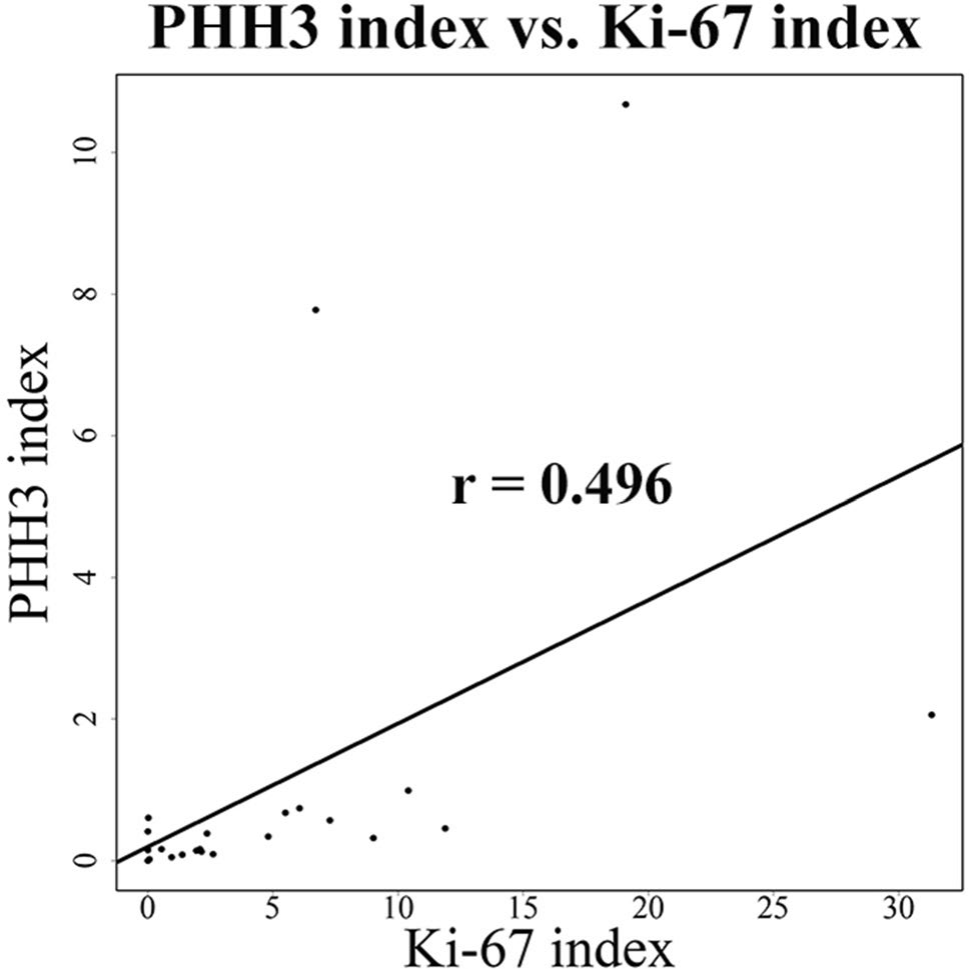
Correlation analysis between PHH3 index and Ki-67 index. Weak positive correlation is indicated.

There was a significant and weak positive correlation between the PHH3 and Ki-67 indices (*P* = 0.01; correlation coefficient = 0.496) (Fig. 4).

### Other factors

Other prognostic factors described in previous reports were investigated, including age, sex, depth, size, surgical margin, the French Fédération Nationale des Centres de Lutte Contre Le Cancer grading system (FNCLCC), necrosis, and proportion of round cell component (RC%). The following cut-off values were based on previously reported literature: age, 60 years; size, 10 cm; RC%, 5%^7,9,10,13–15^. Age and sex demonstrated statistical significance in univariate analysis (*P* ≤ 0.05). A grade of II or more under the FNCLCC system tended to be associated with the poor survival rate (*P* ≤ 0.10) (Fig. 3D). RC% was a significant factor in the multivariate analysis (*P* = 0.02) (Table 4). Depth, size, surgical margin and necrosis did not exhibit statistical significance (Table 5).

**Table 5:** Disease-specific survival rate using the Kaplan–Meier method and univariate analysis using the log-rank test.

Variable	Number of Pt	5-year DSS (%)	*P* value
Age (years)			0.04
< 60	15	92.9	
≥ 60	10	68.6	
Sex			0.03
Female	15	100.0	
Male	10	70.9	
Depth			0.3
Superficial	21	100.0	
Deep	4	80.4	
Tumor size (cm)			0.2
< 10	13	76.2	
≥ 10	12	90.1	
Surgical margin			0.7
R0	21	85.4	
R1	4	66.7	
FNCLCC grading			0.1
grade 1	17	87.4	
≥ grade 2	8	75.0	
Necrosis			0.2
Negative	16	86.5	
Positive	9	77.8	
Round cell component (%)			0.3
< 5	19	88.8	
≥ 5	6	66.7	

*FNCLCC* French Fédération Nationale des Centres de Lutte Contre le Cancer.

## Discussion

In the present study, we demonstrated that the number of PHH3-positive tumor cells and PHH3 index show a significant correlation with the prognosis of MLS patients.

Hendzel et al.^16,17^ reported that the phosphorylation of histone H3 at Ser10 represents a powerful marker for mitotic chromosome condensation in cell proliferation. It is known that PHH3 at Ser10 or Ser28 is specifically expressed during G2 to M phases of a cell cycle, and some authors reported its usefulness as a marker to detect mitotic forms^19^. Fukushima et al.^24^ suggested that PHH3 may be a sensitive and useful marker for meningioma grading based on the mitotic figures in the WHO criteria. Alkhasawneh et al.^25^ presented that PHH3 is associated with inferior overall survival in GIST compared to Ki-67. The effect of immunohistochemistry of PHH3 has also been described in several reports on astrocytoma, melanoma, uterine smooth muscle tumors, pulmonary neuroendocrine carcinomas, and other malignancies^20–23^. In reference to these articles, we focused on PHH3 in MLS patients and investigated the immunohistochemistry of PHH3, in addition to its correlation with prognosis and usefulness as a marker of malignancy compared to other prognostic factors. In this study, we determined a cut-off value of 30 cells per 10 HPF in the number of PHH3-positive tumor cells and 2% in the PHH3 index. Statistically significant differences were found in both criteria by univariate analysis and in the PHH3 index by multivariate analysis, and these significant differences showed their usefulness as predictive factors of progression.

Ki-67 is widely known as an indicator of cell proliferation. This protein exists in the nucleus and is expressed in all phases except G0 of the mitotic cycle^18^. It is used as a predictive factor of tumor prognosis. For example, Pathmanathan et al.^26^ reported that the Ki-67 index was the most powerful and independent predictor of survival in node-negative patients with breast cancer. In this study, a Ki-67 index of over 19% was significantly related to prognosis in MLS patients by univariate analysis. Furthermore, a significant and weak positive correlation was detected between the PHH3 and Ki-67 indices. The weak correlation potentially caused by the result skewed by a few outliers, therefore, if there had been more cases, the correlation may have been higher. Previous studies have also reported a significant correlation between these indices, and the PHH3 index has been identified as a more sensitive predictor of survival^19,24^.

Several prognostic factors of MLS were reported in previous articles. In addition, many authors have considered a range of factors related to prognosis, such as tumor size, histological grade, local recurrence, distant metastasis, age, and sex^7–9,11,14^. The proportion of round cell component, FNCLCC system, Broders’ Grading System, and American Joint Committee on Cancer system (AJCC system) have been adopted to assess histological grade; however, a large body of literature has validated that a statistically significant outcome was obtained with an RC% of greater than 5%^9,10,13,14^. In the current study, age and sex were significant factors in the univariate analysis, and RC% was also significant in the multivariate analysis; however, the FNCLCC system did not show statistical significance.

According to the results of this study, we consider both the PHH3 and Ki-67 indices as useful prognostic factors in MLS. However, the PHH3 and Ki-67 indices present different cut-off values of 2% and 19%, respectively. The use of the PHH3 index is easier than other indices due to its small cut-off value, as the detection of a few positive tumor cells in a single HPF can lead to crossing over the threshold. The PHH3 index may be a more convenient figure than the other in that point.

The main limitation of our study was the small number of patients. Although the long follow-up period and small number of drop-out cases were notable advantages, further studies should be conducted with a larger number of patients. Due to the difficulty of standardization of immunohistochemistry protocols and readout for PHH3 and Ki-67, the current study may have limited generalizability for other laboratories. Standardized methods such as automated immunostaining or artificial intelligence-assisted detection should be considered in the future.

In summary, we examined the histological grade of myxoid liposarcoma, especially the immunohistochemistry of PHH3 and its association with prognosis in this study. The number of PHH3-positive tumor cells, PHH3 index, and Ki-67 index were statistically correlated with the prognosis of MLS. In conclusion, the immunohistochemistry of PHH3 may be associated with prognosis and could serve as a valid criterion of histological grade in MLS. Additional studies about other subtypes of STS are expected in the future.

## Methods

### General information

All medical protocols in this study adhered to the Declaration of Helsinki. This study was approved by the Institutional Review Board of Shinshu University School of Medicine (protocol number: 608) with written informed consent obtained from each participant and/or their legal representative.

### Patient sample

We evaluated 32 patients with MLS who were treated at our hospital from 1995 to 2014. Two patients with unknown progression status and 5 patients who underwent non-surgical treatment were excluded. After these exclusions, the remaining 25 patients were included in the study. FUS-DDIT3/EWSR1-DDIT3 fusion was confirmed by fluorescence in situ hybridization testing in 5 patients who were treated since 2010.

### Immunohistochemistry

Formalin-fixed and paraffin-embedded surgical specimens were prepared in cross sections. Sections of specimens with the highest cell density and rich atypical cells were selected. For immunohistochemistry, mouse monoclonal anti-Ki-67 (clone MIB-1) and rabbit polyclonal anti-PHH3 (Cell Marque, cat. no. 369A-15) antibodies were purchased from Dako (Glostrup, Denmark) and Merck (Darmstadt, Germany), respectively. Antigen retrieval for Ki-67 antigen was carried out by heating tissue slides in 10 mM Tris–HCl buffer (pH 8.0) and 1 mM EDTA with a microwave oven for 30 min (for Ki-67) or a pressure cooker for 10 min (for PHH3). As secondary antibodies, a Histofine Simple Stain MAX-PO (M) Kit (Nichirei Biosciences, Tokyo, Japan) was used for Ki-67 antigen, and a Histofine Simple Stain MAX-PO (R) (Nichirei Biosciences) was used for PHH3 antigen. Peroxidase activity was visualized using a diaminobenzidine/H_2_0_2_ solution. Counterstaining was conducted using a Carrazzi's Hematoxylin solution (× 2) prepared from hematoxylin (H9627; Sigma-Aldrich, MO, USA). As an on-slide control, tumor cells in the tissue slides were used for positive control. Negative control immunohistochemistry was performed by omitting the primary antibodies from the procedure, and no specific staining was observed. This procedure was determined to be adequate, because the positive and negative controls were stained precisely.

### Assessment of PHH3 and Ki-67

In both PHH3 and Ki-67 immunohistochemistry, a tumor cell was determined to be positive when more than 70% of the nuclear area of the tumor cell was immunostained dark brown (Supplemental Fig. 1). According to previous reports^19,20,22,24^ the number of total tumor cells and positive tumor cells on the hot spots of each specimen were manually measured in 10 HPF by a surgical oncologist (Ak.T.) and confirmed by a pathologist (J.N.). The positive rates of PHH3 and Ki-67 were subsequently calculated for consideration as PHH3 and Ki-67 indices. Both were measured by the same method for comparison. The FNCLCC grading, necrosis, and round cell component were evaluated on hematoxylin and eosin (H & E) specimens of the entire cross section by pathologists (T.U. and J.N.).

### Statistical analysis

Prognostic factors of the examination criteria were statistically analyzed. DSS was also evaluated by defining DOD as the endpoint. The cut-off values regarding DOD were determined using an ROC curve for the number of PHH3 positive cells, PHH3 and Ki-67 indices. Other items deemed to be common were based on previous reports. Survival analyses were performed using the Kaplan–Meier method, and univariate analyses were performed using the Log-rank test. The multivariate analysis was performed using the Cox proportional-hazard regression analyses with the PHH3 index, Ki-67 index, and RC% as explanatory variables. Statistical significance was defined as a *P*-value of 0.05 or less. The software R version 4.0.3 was used for analyses.

## Supplementary Information

Supplementary Information 1.

Supplementary Table 2.

## Data Availability

The data collected and analyzed during this study are available from the corresponding author upon reasonable request.
