# Supplementary figures and images for: The usefulness of immunohistochemistry for phosphohistone H3 as a prognostic factor in myxoid liposarcoma
Source: Sci Rep. 2023 Mar 23;13:4733. doi: 10.1038/s41598-023-31896-y (PMC10036607; doi:10.1038/s41598-023-31896-y)

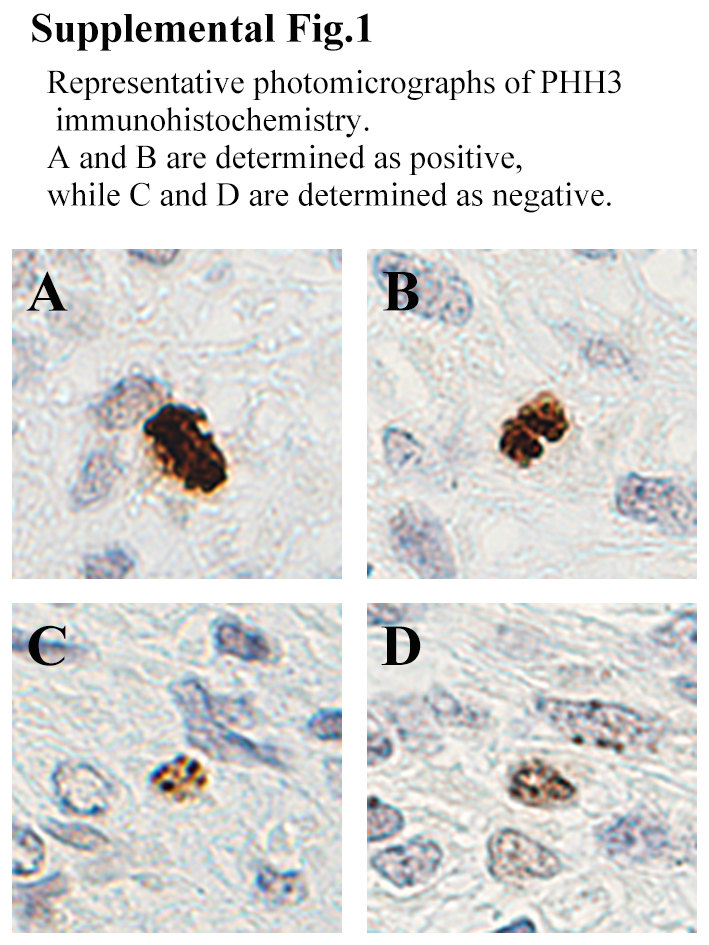

Supplement: Supplementary file 1 — Supplementary Information 1. [file 41598_2023_31896_MOESM1_ESM.jpg]
