# Supplementary material for: The usefulness of immunohistochemistry for phosphohistone H3 as a prognostic factor in myxoid liposarcoma
Source: Sci Rep. 2023 Mar 23;13:4733. doi: 10.1038/s41598-023-31896-y (PMC10036607; doi:10.1038/s41598-023-31896-y)
Supplement: Supplementary file 2 — Supplementary Table 2. [file 41598_2023_31896_MOESM2_ESM.pdf]

Supplemental Table 1

Clinicopathological and immunohistochemical data of each individual patient with myxoid liposarcoma

Patient No.	Age (years)	Sex	Follow up (years)	Depth	Tumor size (cm)	Surgical margin	FNCLCC grading	Necrosis	Round cell component (%)	All cells (/10HPF)	PHH3 positive tumor cells (/10HPF)	Ki-67 positive tumor cells (/10HPF)	PHH3 index (%)	Ki-67 index (%)	Outcome
1	53	M	4.5	D	6	R1	1	N	0	286	31	54	10.7	19.1	DOD
2	54	F	10.3	D	8	R0	1	P	0	5522	6	144	0.1	2.2	CDF
3	75	M	6.3	D	4.4	R0	2	P	0	2959	38	984	2.1	31.3	DOD
4	84	M	1.5	D	6.6	R0	1	N	0	750	45	43	7.8	6.7	DOD
5	50	M	5.9	D	10.4	R0	3	N	5	2852	27	339	1.0	10.4	CDF
6	44	M	5.4	D	13.5	R0	3	P	5	4660	23	598	0.5	11.9	CDF
7	57	F	9.8	D	12	R0	1	N	0	1203	1	17	0.1	1.4	CDF
8	53	M	4.0	D	8.1	R0	1	N	0	321	4	19	0.7	5.5	CDF
9	64	M	4.4	D	23	R0	2	P	10	2463	13	230	0.3	9.0	DOD
10	31	F	9.0	D	6.7	R0	1	N	0	2119	12	177	0.6	7.3	CDF
11	31	M	6.8	D	15.8	R1	2	N	5	4659	1	3	0.0	0.1	AWD
12	67	F	8.7	S	4.5	R0	1	N	0	2543	4	55	0.1	1.9	CDF
13	37	F	11.6	S	6.2	R0	1	N	0	1683	3	0	0.2	0.0	CDF
14	50	F	11.9	D	12	R0	1	N	0	2058	3	11	0.2	0.6	CDF
15	41	M	10.3	D	5	R0	1	N	0	868	4	0	0.4	0.0	CDF
16	43	F	12.4	D	7.6	R0	2	P	0	2445	2	77	0.1	2.6	CDF
17	40	F	10.6	S	9	R0	2	P	0	3373	5	84	0.1	2.1	CDF
18	60	M	1.9	D	9	R0	2	P	50	5024	8	0	0.1	0.0	DOD
19	39	F	22.3	D	20.5	R0	2	P	20	2908	18	1	0.6	0.0	CDF
20	66	M	9.6	D	8.6	R1	1	N	0	1282	1	8	0.0	1.0	CDF
21	49	M	8.2	D	16	R0	1	N	0	283	0	0	0.0	0.0	CDF
22	68	M	4.3	S	10	R1	1	N	0	2385	8	131	0.3	4.8	DOOD
23	75	F	6.5	D	22	R0	1	N	0	2558	4	53	0.2	2.1	DOOD
24	62	M	15.3	D	13	R0	1	N	0	2694	20	191	0.7	6.1	CDF
25	85	M	7.0	D	13	R0	1	N	0	2602	10	60	0.4	2.4	CDF

FNCLCC: French Fédération Nationale des Centres de Lutte Contre Le Cancer; HPF: high power fields; PHH3: phosphohistone H3; CDF: continuous disease free; AWD: alive with disease; DOD: dead of disease; DOOD: dead of other disease; M: Male; F: female; S: superficial; D: deep; P: positive; N: negative.
